# Analysis of vitamin D level among asymptomatic and critically ill COVID-19 patients and its correlation with inflammatory markers

**DOI:** 10.1038/s41598-020-77093-z

**Published:** 2020-11-19

**Authors:** Anshul Jain, Rachna Chaurasia, Narendra Singh Sengar, Mayank Singh, Sachin Mahor, Sumit Narain

**Affiliations:** 1 Department of Anaesthesiology, M.L.B Medical College, Jhansi, India; 2 Department of Radiodiagnosis, M.L.B Medical College, Jhansi, India; 3 Department of Nephrology, M.L.B Medical College, Jhansi, India; 4 Department of Pathology, M.L.B Medical College, Jhansi, India; 5 Department of Radiotherapy, COVID-19 Block M.L.B Medical College, Jhansi, India

**Keywords:** Microbiology, Endocrinology, Health care, Risk factors

## Abstract

COVID-19 is characterized by marked variability in clinical severity. Vitamin D had recently been reviewed as one of the factors that may affect the severity in COVID-19. The objective of current study is to analyze the vitamin D level in COVID-19 patients and its impact on the disease severity. After approval from Ethics Committee, M.L.B Medical College the current study was undertaken as continuous prospective observational study of 6 weeks. Participants were COVID-19 patients of age group 30–60 years admitted during the study period of 6 weeks. Study included either asymptomatic COVID-19 patients (Group A) or severely ill patients requiring ICU admission (Group B). Serum concentration of 25 (OH)D, were measured along with serum IL-6; TNFα and serum ferritin. Standard statistical analysis was performed to analyze the differences. Current Study enrolled 154 patients, 91 in Group A and 63 patients in Group B. The mean level of vitamin D (in ng/mL) was 27.89 ± 6.21 in Group A and 14.35 ± 5.79 in Group B, the difference was highly significant. The prevalence of vitamin D deficiency was 32.96% and 96.82% respectively in Group A and Group B. Out of total 154 patients, 90 patients were found to be deficient in vitamin D (Group A: 29; Group B: 61). Serum level of inflammatory markers was found to be higher in vitamin D deficient COVID-19 patients viz. IL-6 level (in pg/mL) 19.34 ± 6.17 vs 12.18 ± 4.29; Serum ferritin 319.17 ± 38.21 ng/mL vs 186.83 ± 20.18 ng/mL; TNFα level (in pg/mL) 13.26 ± 5.64 vs 11.87 ± 3.15. The fatality rate was high in vitamin D deficient (21% vs 3.1%). Vitamin D level is markedly low in severe COVID-19 patients. Inflammatory response is high in vitamin D deficient COVID-19 patients. This all translates into increased mortality in vitamin D deficient COVID-19 patients. As per the flexible approach in the current COVID-19 pandemic authors recommend mass administration of vitamin D supplements to population at risk for COVID-19.

## Introduction

In December 2019, several cases of pneumonia with unknown etiology reported in Wuhan, Hubei Province, China^1,2^. The disease spread quickly to other provinces of China and overseas. On 7 January 2020, a novel coronavirus was identified in the throat swab sample of one such patient and later declared to be the etiologic virus and was subsequently named as 2019nCoV by World Health Organization (WHO)^3^. On worsening of the situation WHO declared the outbreak as the public health emergency of international concern (PHEIC). In February 2020, WHO provided a nomenclature to the epidemic disease caused by SARS-CoV-2 as coronavirus disease 2019 (COVID-19)^4^. As on 12th August 2020 there are more than 20 million cases worldwide^5^, so for now it’s almost impossible to contain the disease spread and focus is diverting towards better treatment and prevention of factors that enhance the severity of COVID-19. COVID-19 is characterized by its high infectivity and marked variability in clinical severity, of which 40–45% patients remain asymptomatic and 30–40% develop only mild symptoms. Only fewer than 15% of cases develop severe disease^6^. Diabetes and hypertension are commonest co morbidities associated with severe disease^7^. Until recently researchers focus towards more deep survey of the modifiable factors which enhance/reduce the severity of COVID-19. Authors after reviewing the literature available postulated the hypothesis that vitamin D level plays significant role in determining the severity of COVID-19.

Vitamin D known to play key role in the maintenance of bone health and calcium–phosphorus metabolism, yet many other functions of this vitamin have been recently postulated, such as modulation of the immune response in both infectious and autoimmune diseases^8,9^. Vitamin D includes fat soluble secosteroids that are responsible for a wide spectrum of immunomodulatory, anti-inflammatory antifibrotic, and anti-oxidant actions. In humans vitamin D_3_ (cholecalciferol) and vitamin D_2_ (ergocalceferol) are the most abundant subtype of vitamin D. Liver converts vitamin D_3_ in to calcifediol (25-hydroxycholecalciferol); and D_2_ subtype is converted into 25-hydroxyergocalciferol. 25-hydroxyvitamin D or 25(OH)D) the principal metabolite of these two vitamin D can be measured in serum to know the vitamin D status of the individual^10,11^. Calcitriol (1,25-(OH)_2_D), is the active form of vitamin D which is generated by 1α hydroxylase enzyme present in kidney^12^. Calcitriol circulates as a hormone in the blood, playing major role in calcium and phosphate homeostasis and encourages the healthy remodeling of the bone. Beside this calcitriol, has definite role in cellular growth, neuromuscular functions and plays an important role in immune functions, in particular with anti-inflammatory action. It inhibits the expression of inflammatory cytokine [e.g., IL-1α, IL-1β, tumor necrosis factor-α] and its insufficiency was associated with over- expression of Th1 cytokines^8,13^.

To confirm the hypothesis authors performed the present observational study in which authors measured the vitamin D levels of all COVID-19 patients who had been advised admission in COVID-ICU and simultaneously in COVID -19 patients who were asymptomatic. The interleukin 6 (IL-6), tumor necrosis factor α (TNFα) and ferritin concentration was also measured in all patients and correlated with serum 25(OH) D concentration.

## Material and methods

### Study design and subjects

The current study was undertaken as continuous prospective observational study of 6-week duration. All experimental protocols were approved by Ethics Committee M.L.B Medical College. An informed and written consent was obtained either from the participants himself or their first degree relative. Participants were COVID-19 patients of age group 30–60 years who were admitted in tertiary COVID-19 care center during the study span of 6 weeks. First participant was recruited on 5th June. Only two subtypes of COVID-19 patients were included in study.

#### Group A

RT-PCR confirmed COVID-19 patients who were asymptomatic at the time of admission and remained asymptomatic till discharge on 12th Day.

#### Group B

RT-PCR confirmed COVID-19 patients requiring ICU admission due to severe COVID disease.

#### Sample size

Authors included all eligible subjects admitted to dedicated COVID-19 management center during study span of 6 weeks started from 5th June 2020 and all eligible subjects were followed till the closure of case i.e. either discharge or mortality.

#### Exclusion criteria

Pregnancy; chronic obstructive airway disease; chronic renal disease patients on dialysis, patients on chemotherapy were excluded from the study.

Institute adopts following criteria for ICU admission of COVID-19 patients:

- Clinical signs of pneumonia (fever, cough, breathlessness) plus one of the following: respiratory rate > 30 breaths/min; severe respiratory distress; or SpO_2_ < 90% on room air.
- Signs of multi-organ involvement: altered sensorium, decreased urine output, heart Rate > 120/min, with cold extremities or low blood pressure (Systolic BP < 90 mm of Hg and/or Diastolic BP < 60 mm of Hg).
- Laboratory evidence of coagulation abnormalities, thrombocytopenia, acidosis (pH < 7.25), lactate level > 2 mmol/L, or hyperbilirubinemia.

### Intervention and evaluation

All COVID-19 patients who got admitted in the recruitment period of 6 weeks starting from 5th June were evaluated at triage and advised admission to dedicated COVID wards accordingly. Asymptomatic and mildly symptomatic patients were advised admission in isolation wards. Patients with moderate and severe COVID-19 were advised admission in high dependency unit. Patients with severe COVID-19 who fulfilled ICU admission criteria were advised admission in ICU. Asymptomatic patients were allotted to Group A and given serial number series A1, A2, A3 and so on where as ICU patients were allotted to Group B and given number B1, B2, B3 and so on.

After informed and written consent Serum 25 (OH)D estimation was performed along with other routine blood test that includes complete blood count; liver function test; renal function test and estimation of Serum IL-6; TNFα and ferritin level. On the basis of multiple guidelines serum 25(OH) D level of < 20 ng/mL has been defined as vitamin D deficiency^14^. Calculations were also performed adopting 30 ng/ml as the cutoff level of 25(OH) D for defining vitamin D deficiency^15^. Serum 25 (OH)D concentration was estimated by automated immunoassays through Architecti1000sr Make 2015 last calibration on 2nd June 2020. All investigations were carried out in accordance with relevant guidelines and regulations.

### Statistical analysis

Quantitative data are expressed as mean ± 2SD. Qualitative variables were expressed as proportions. Serum 25 (OH)D concentration was expressed as continuous variable. Cumulative prevalence of vitamin D deficiency estimated in both the groups using standard formula. Mann–Whitney *U* test; unpaired ‘t’ test and Chi Square Test was performed for statistical analysis. *p*-value less than 0.05 was considered statistically significant. SPSS for Windows (version 22; IBM SPSS Inc., Chicago IL) was used for all statistical analyses.

## Results

**Figure 1:**
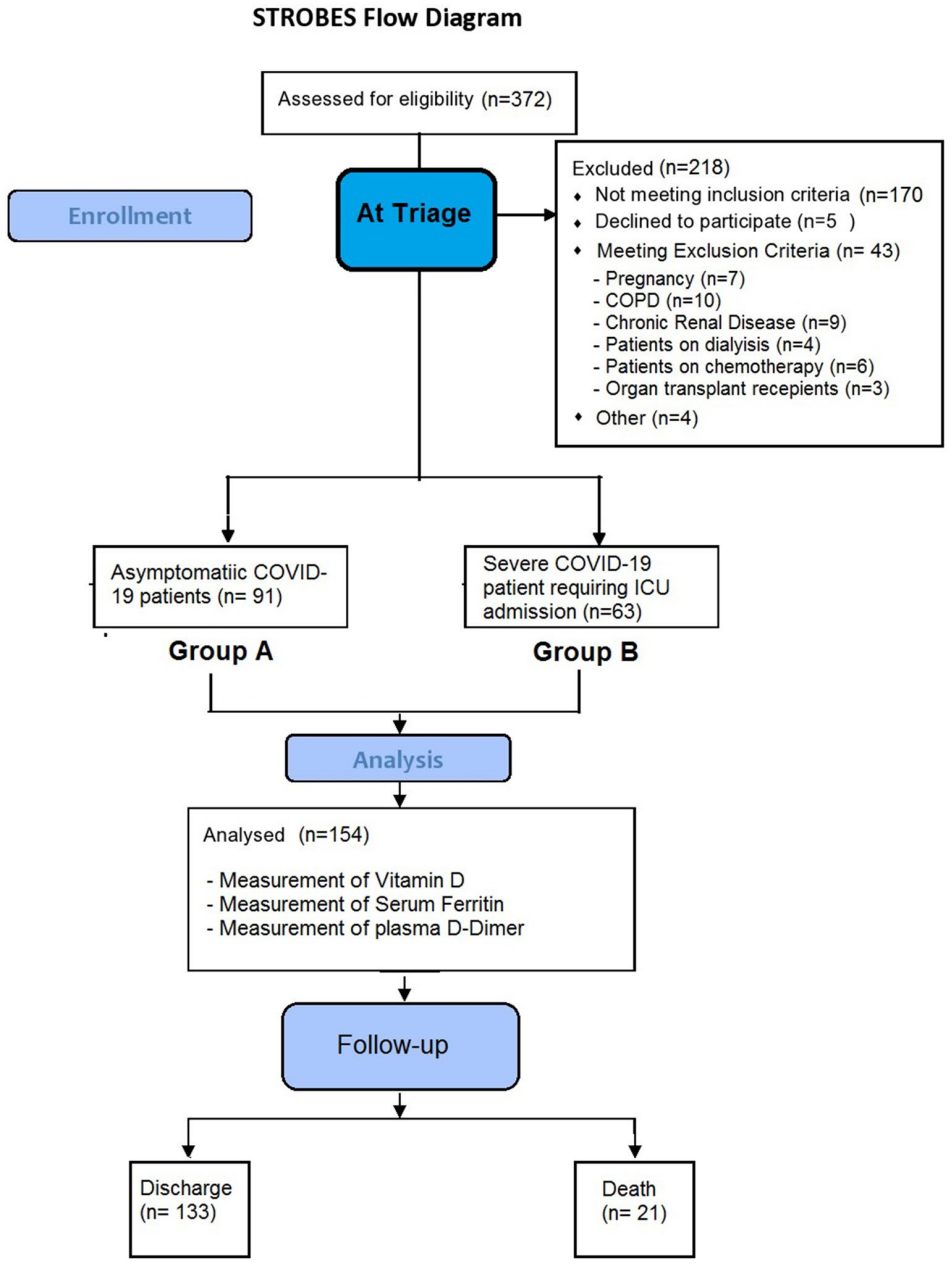
Strobes flow diagram.

372 COVID-19 patients were admitted in the institute in the study span of six weeks, out of which 202 patients were enrolled in current study after applying inclusion criteria. On 48 patients were excluded after application of exclusion criteria providing the final number of 154 patients as shown in Strobes flow diagram (Fig. 1). Out of 154 patients 91 were asymptomatic (Group A) 63 patients were severely ill and had required ICU admission (Group B).

The demographic variables are shown in Table 1 and there was statistically significant difference in the mean age and sex distribution. Among patients admitted in ICU the mean age was higher and sex ration predominated towards male sex.

**Table 1:** Demographic distribution of subjects.

Parameter	Group A (n = 91)	Group B (n = 63)	‘p’ value
Male: female	53:48	42:21	0.03*
Age (in years) (mean ± 2SD)	42.34 ± 6.41	51.41 ± 9.12	0.01**
Weight (in Kg) (Mean ± 2SD)	67.18 ± 12.34	64.29 ± 11.67	0.09*
Height (in meters) (Mean ± 2SD)	1.12 ± 0.34	1.10 ± 0.41	0.1*
Body Mass Index (Kg/m^2^) (Mean ± 2SD)	27.23 ± 3.45	26.83 ± 5.81	0.09*

Group A: Asymptomatic COVID-19 patients, Group B: Critically ill COVID-19 patients.

*Un-paired t Test; **Chi Square test.

**Figure 2:**
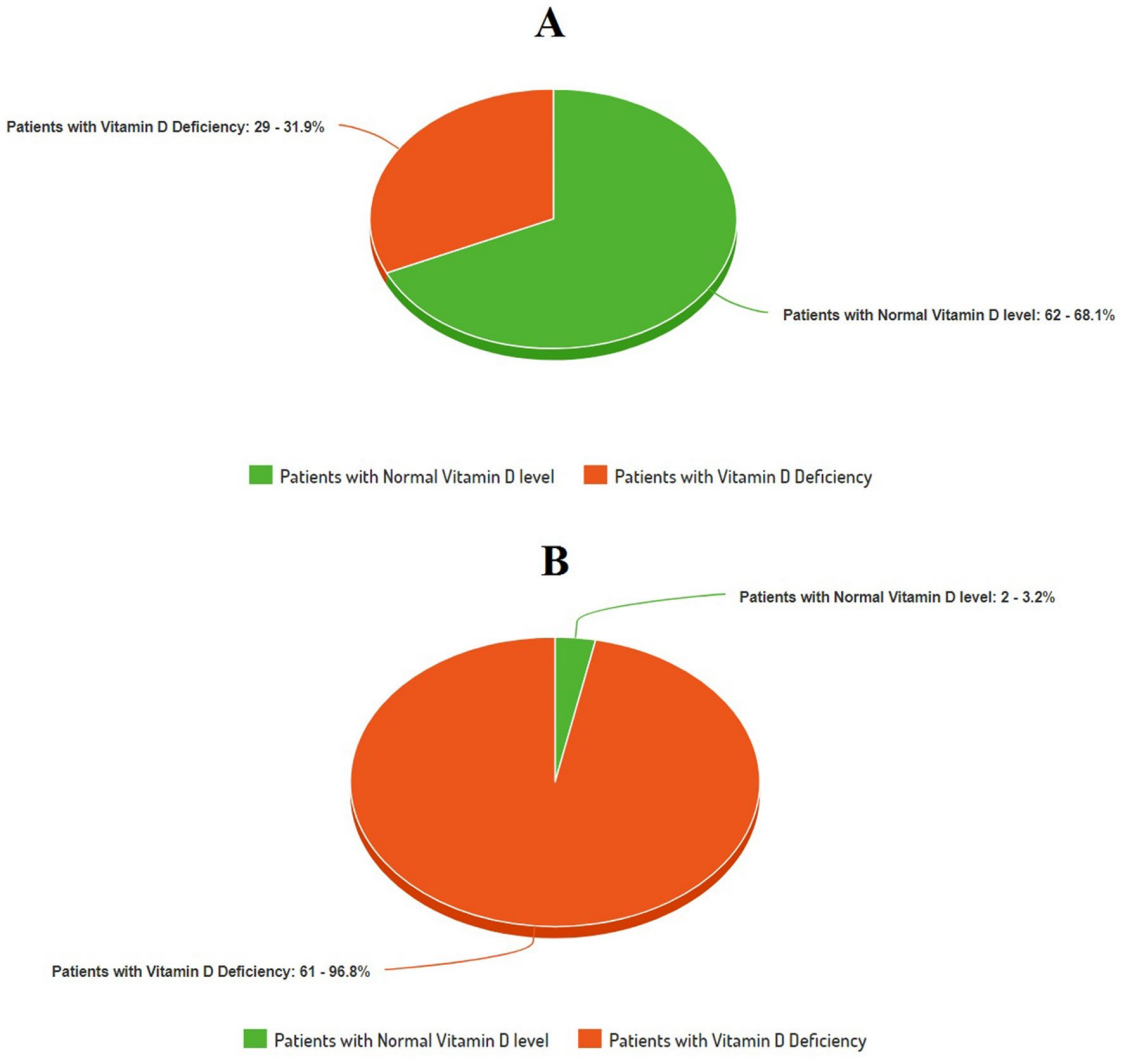
Prevalence of vitamin D deficiency.

The mean concentration (in ng/mL) of 25 (OH)D in Group A was 27.89 ± 6.21where as in Group B the mean level was 14.35 ± 5.79. (Table 2) When compared statistically the difference was found to be highly significant. The prevalence of vitamin D deficiency was 31.86% in Group A. In Group B 96.82% patients were vitamin D deficient (Fig. 2). On Chi square test the difference in the prevalence of vitamin D among two groups was found to be highly significant. On adopting the cutoff concentration level of Serum 25 (OH) D as < 30 ng/ml for defining vitamin D deficiency the prevalence of vitamin D deficiency was 43.95% in Group A and 98.41% in Group B.

**Table 2:** (A) Vitamin D and Serum Ferritin level in Group A and Group B, (B) Relationship of inflammatory markers with serum 25 (OH) D level as continuous variable).

Parameter	Group A (n = 91)	Group B (n = 63)	p value (group A vs group B)
**(A) Comparison of 25 (OH) D level in asymptomatic COVID-19 patients (Group A) and critically ill COVID-19 patients (Group B)**			
Vitamin D (Serum 25 (OH) D level) in ng/mL (mean ± 2SD)	27.89 ± 6.21	14.35 ± 5.79	0.0001*
Serum Ferritin in ng/mL (mean ± 2SD)	198.23 ± 23.16	331.68 ± 36.41	0.0003*
**(B) Serum 25 (OH) D level as continuous variable**			
	1st Tertile (n = 51, 3.2–9.1 ng/mL)	2nd Tertile (n = 51, 9.2–27.7 ng/mL)	3rd Tertile (n = 52, 27.8–49.1 ng/mL)
Group A (n = 91)	12	27	52
Group B (n = 63)	39	23	1
Serum Ferritin in ng/mL (mean ± 2SD)	384.22 ± 27.34	231.45 ± 18.14	169.98 ± 17.43
Serum IL-6 in pg/mL (mean ± 2SD)	21.23 ± 5.18	17.35 ± 3.49	10.39 ± 4.47
Serum TNFα in pg/mL (mean ± 2SD)	14.26 ± 6.78	12.59 ± 3.91	10.49 ± 5.12

Group A: Asymptomatic COVID-19 patients, Group B: Critically ill COVID-19 patients.

*Mann–Whitney *U* test.

On categorization of the patients on the basis of vitamin D Deficiency, out of total 154 patients, 90 patients were found to be deficient in vitamin D among which 61 were critical and 29 were asymptomatic (Table 3). If we take 10 ng/mL as the guide for severe vitamin D deficiency, 62 patients were found to be severely deficient in vitamin D among which 52 were critical and 10 were asymptomatic. Two critical patients have normal level of vitamin D.

**Table 3:** Inflammatory markers in relation to Vitamin D.

Parameter	Patients with (Serum 25 (OH) D level) > 20 ng/mL n = 64 [X]	Patients with (Serum 25 (OH) D level) < 20 ng/mL n = 90 [Y]	‘p’ value [X] vs [Y]
Group A	62	29	NA
Group B	2	61	NA
Serum IL-6 in pg/mL (mean ± 2SD)	12.18 ± 4.29	19.34 ± 6.17	0.03*
Serum TNF α in pg/mL (mean ± 2SD)	11.87 ± 3.15	13.26 ± 5.64	0.06*
Serum ferritin in ng/mL (mean ± 2SD)	186.83 ± 20.18	319.17 ± 38.21	0.0003*

Group A: Asymptomatic COVID-19 patients; Group B: Critically ill COVID-19 patients.

*Chi-square test.

*SD* Standard deviation.

The analysis of serum level of inflammatory markers reveals mean IL-6 level (in pg/mL) of 19.34 ± 6.17 in patients with vitamin D deficiency (serum Serum 25 (OH)D < 20 ng/mL) and 12.18 ± 4.29 in patients with normal vitamin D level, the difference was found to be statistically significant. Serum ferritin level was also significantly high in patients with vitamin D deficiency (319.17 ± 38.21 ng/mL vs 186.83 ± 20.18 ng/mL) than in patients with normal vitamin D. Serum TNFα level (in pg/mL) was also high in vitamin D deficient COVID-19 patients (13.26 ± 5.64 vs 11.87 ± 3.15) but the difference was not significant (Table 3).

Among both the groups diabetes was the commonest co-morbidity followed by hypertension. On follow up till closure of case (discharge vs fatality), the fatality rate in Group A was 1.09% (1 patient died) whereas in Group B was 31.74% (20 patients died). When the fatality was compared on the basis of vitamin D deficiency, the fatality rate was 21% (19 patients died in 90 patients) among vitamin D deficient and 3.1% (2 patient died in 64) among patients with normal vitamin D level.

## Discussion

The clinical manifestations of COVID-19 varies from asymptomatic or paucisymptomatic forms to critical illness characterized by respiratory failure that warrants mechanical ventilation in an ICU^6^. Multiple organ dysfunction syndromes (MODS); sepsis and septic shock are other serious COVID-19 manifestation that warrants ICU admission. As per various studies only 10–15% of cases develop serious disease. Even in same age group without any cormorbid condition there is wide variation in the clinical severity. As till now there is no definitive treatment or vaccine is available for SARS-CoV-2., if one can identify some or other modifiable factor whose presence or absence affects the severity of disease one can definitely reduce the severity of disease. The first such factor that comes to mind is viral load itself. However, Argyropoulos et al. ruled out any association of initial viral load to the severity or chances of developing acute respiratory distress syndrome in SARS-CoV-2 patients^16^. There were media reports which correlates severity of COVID-19 with blood group, however the prospective multi-institutional study conducted by Latz et al. ruled out any independent association between blood type and peak inflammatory markers. The study concluded that no specific blood type is associated with the risk of intubation or mortality in COVID-19^17^.

Regarding, acute lung injury in COVID-19 the data so far available has indicated than an unrestricted immune reaction in the host is the main process which leads to so called 'cytokine storm' the net effect is is extensive tissue damage with dysfunctional coagulation^18,19^. Just a month ago, Italian researchers introduced MicroCLOTS (microvascular COVID-19 lung vessels obstructive thromboinflammatory syndrome) as mechanism for underlying pulmonary injury in COVID-19^20^. Out of several cytokines like the tumor necrosis factor α (TNF-α), IL-1β, IL-8, IL-12 play definite role in the pathogenic cascade of the disease and the most important mediator of this storm is interleukin 6 (IL-6)^19,21^. IL-6 can be produced by immune system cells (B lymphocytes, T lymphocytes, macrophages, dendritic cells, monocytes, mast cells); stromal cells and by many non-lymphocytes cells including fibroblast and endothelial cells^22^. IL-1β and TNFα are the key activators for the secretion of the IL-6^23^.

Vitamin D is usually acknowledged for the maintenance of bone health and calcium–phosphorus metabolism, many other roles like stimulation of insulin production, effects on myocardial contractility have been recently discovered. Vitamin D plays an essential role in the immune system. Vitamin D interferes with the majority of the immune systems cells such as macrophages, B and T lymphocytes, neutrophils and dendritic cells^24^. The T and B lymphocytes can form the active metabolite of vitamin D, 1,25(OH)2D3 which inhibits T cell proliferation and activation. Beside this, vitamin D inhibits the production of pro-inflammatory cytokines and enhance the production of anti-inflammatory cytokines^25,26^. Vitamin D inhibits the adaptive immune system and promotes the innate immune system which balances the immune response and provides an overall anti-inflammatory response^27^.

In Current study authors found that vitamin D deficiency (as suggested by serum 25 (OH)D concentration < 20 ng/mL) is far more prevalent in patients with severe COVID–19 disease requiring ICU admission and thereby increased chances of mortality. For non-skeletal purpose many researchers had suggested cutoff level of serum 25 (OH)D to < 30 ng/ml for defining vitamin D deficiency^15^, on adopting this criterion the prevalence was almost 100% in critically ill patients (62 out of 63). On the same side the patients with vitamin D deficiency exhibit higher levels of chemical markers of inflammation. The current study is the first and the most comprehensive study in which both severe and asymptomatic COVID patients were included and vitamin D level along with inflammatory markers were estimated the so as to correlate the association. In current study authors preferred of using period-based inclusion of subject over specifying the sample size because of the reason that COVID-19 is an emerging pandemic with variable level of seropositivity in the society and none of the sample size formula fits well with satisfactory reduction in the chances of error. Authors adopted 6-week criterion for inclusion of eligible subjects which is around 10.71% of year (56 weeks). Beside this all the subjects were followed till the closure that is successful discharge or mortality.

In western countries there is a strong association of vitamin D deficiency with socioeconomic status, which doesn’t holds true in India, rather few studies have demonstrated lower prevalence of vitamin D deficiency in low socioeconomic status and correlated this to higher sunlight exposure in lower socioeconomic strata of India^28^. Keeping this in view authors excluded accounting of socioeconomic status as independent variable.

Jun Xu, et al. in had demonstrated beneficial effect of vitamin D agonist, calcitriol, on LPS-induced acute lung injury in rats; they had demonstrated that calcitriol pretreatment significantly improved LPS-induced lung permeability. Through ELISA analysis they demonstrated that calcitriol modulates the expression of members of the renin-angiotensin system (RAS), including angiotensin I-converting enzymes (ACE and ACE2), renin and angiotensin II, to exert the protective effects on LPS-induced lung injury^29^. Surprisingly, SARS Cov-2 also uses ACE receptors for infection.

Biesalski, in the review article emphasize the fatal relationship of Vitamin D and comorbidities in COVID-19 patients^30^. E Laird, J Rhodes also conducted a literature search study to conclude that optimising vitamin D status certainly have benefits in COVID-19^31^.

The results of current study however be interpreted with few limitations. First, the study has been conducted in a single centre located in central India, the area has itself high prevalence of vitamin D deficiency. Second: Time elapsed between actual infection and admission has not been taken in to consideration and the quantitative variables are measured only when patient got admitted in the hospital. This may have impact over chemical markers of inflammation. Third Current study doesn’t take co morbidities in account while estimating pro-inflammatory markers as co-morbidities like diabetes and hypertension enhance the severity in COVID-19. So keeping these in view a multicentre study with large number of subjects can be carried out or a large pooled prospective datasets can be collected to re-assess the result and will generate more robust conclusions.

## Conclusion

Vitamin D deficiency markedly increases the chance of having severe disease after infection with SARS Cov-2. The intensity of inflammatory response is also higher in vitamin D deficient COVID-19 patients. This all translates to increase morbidity and mortality in COVID-19 patients who are deficient in vitamin D. Keeping the current COVID-19 pandemic in view authors recommend administration of vitamin D supplements to population at risk for COVID-19.
